# Association of baseline inflammatory biomarkers with cancer mortality in the REGARDS cohort

**DOI:** 10.18632/oncotarget.27108

**Published:** 2019-08-06

**Authors:** Tomi Akinyemiju, Justin X. Moore, Maria Pisu, Michael Goodman, Virginia J. Howard, Monika Safford, Susan C. Gilchrist, Mary Cushman, LeAnn Long, Suzanne E. Judd

**Affiliations:** ^1^ Department of Population Health Sciences, Duke University School of Medicine, Durham, NC, USA; ^2^ Division of Public Health Sciences and Siteman Cancer Center, Washington University School of Medicine, St. Louis, MO, USA; ^3^ Division of Preventive Medicine, University of Alabama at Birmingham, Birmingham AL, USA; ^4^ Department of Epidemiology, Emory University Rollins School of Public Health, Atlanta, GA, USA; ^5^ Department of Epidemiology, University of Alabama at Birmingham, Birmingham AL, USA; ^6^ Department of Medicine, Weill Cornell Medical College, New York, NY, USA; ^7^ Department of Clinical Cancer Prevention and Cardiology, The University of Texas MD Anderson Cancer Center, Houston, TX, USA; ^8^ Department of Medicine and Vermont Cancer Center, Larn er College of Medicine at the University of Vermont, Burlington, VT, USA; ^9^ Department of Biostatistics, University of Alabama at Birmingham, Birmingham, AL, USA

**Keywords:** inflammatory biomarkers, racial disparities, cancer mortality, obesity

## Abstract

This study examines the association between inflammatory biomarkers and risk of cancer mortality by race. Data were obtained from 1,856 participants in the prospective REGARDS cohort who were cancer-free at baseline, and analyzed in relation to cancer mortality prospectively. Biomarkers were log-transformed and categorized into tertiles due to non-normal distributions, and Cox proportional hazard regression models were utilized to compute hazard ratios with 95% confidence intervals using robust sandwich methods. Individuals in the highest tertile of IL-6 had over a 12-fold increased risk of cancer mortality (HR: 12.97, 95% CI: 3.46–48.63); those in the highest tertile of IL-8 had over a 2-fold increased risk of cancer mortality (HR: 2.21, 95% CI: 0.86–5.71), while those in the highest tertile of IL-10 had over a 3-fold increased risk of cancer mortality (HR: 3.06, 95% CI: 1.35–6.89). In race-stratified analysis, each unit increase in IL-6 was associated with increased risk of cancer mortality among African-Americans (HR: 3.88, 95% CI: 1.17–12.88) and Whites (5.25, 95% CI: 1.24–22.31). If replicated in larger, racially diverse prospective cohorts, these results suggest that cancer patients may benefit from clinical or lifestyle approaches to regulate systemic inflammation as a cancer prevention strategy.

## INTRODUCTION

The link between inflammation and cancer development and progression is well known, and while inflammation-associated cell proliferation alone does not cause cancer, sustained cell proliferation in a highly enriched inflammatory environment promotes tumorigenesis [1]. Epidemiologic studies reveal that long-term aspirin and nonsteroidal anti-inflammatory drug use is associated with up to 40–50% reduced risk of colon cancers [2], 45% reduced risk of pre-menopausal breast cancer [3], and reduced risk of lung, esophagus and stomach cancers [4]. Other studies have shown that higher systemic inflammation significantly increases risk of cancer-related mortality [5–7]. The molecular mechanisms explaining the association of inflammation and cancer risk have been the subject of much research [8–12]; however, questions remain regarding the role of specific cytokines as predictors of cancer mortality. Biomarkers of chronic inflammation [13] and cancer mortality rates vary significantly by race in the U.S [14], likely due to differences in socioeconomic status, behavioral factors such as diet and exercise, as well as access to quality healthcare [15–19]. Obesity is also an established predictor of cancer risk and mortality [20, 21], and recent studies including by our group [22], suggests that obesity-associated chronic inflammation may be an important prognostic risk factor for cancer mortality [23, 24]. CRP, an acute-phase protein synthesized in response to systemic inflammation, has also been well studied as a marker of overall inflammatory response [25], however it has not been well characterized in the context of cancer mortality disparities. Inflammation-related cytokines are easily measured from blood samples, and if shown to be independently associated with cancer mortality, may be useful as prognostic biomarkers for cancer. If racial differences exist in these associations, it may enhance understanding of the biological basis of racial disparities in cancer mortality. Few studies to date have examined the role of baseline circulating inflammatory cytokines and long-term risk of cancer mortality in a racially diverse prospective cohort. In the present study, we examined whether baseline biomarkers of inflammation increase the risk of overall and obesity-related cancer mortality among REGARDS participants overall and stratified by race.

## RESULTS

A total of 1,856 participants and 86 cancer deaths were observed during the study period with average follow up time of 6.5 (SD = 2.2) years, and about 32% of the cancer deaths were for obesity-related cancers. Higher baseline IL-6 was associated with older age, African-American race (51% vs. 35%, *p <* 0.01), lower educational attainment (18% vs. 7%, *p <* 0.01), lower income (23% vs. 11%, *p <* 0.01), and greater proportion of current smokers (23% vs. 11%, *p <* 0.01), Table 1. Higher baseline IL-8 was associated with older age, lower educational attainment (13% vs. 10%, *p <* 0.01), current smoking (17% vs. 11%, *p <* 0.01) and higher statin use (39% vs. 31%, *p <* 0.01), and higher baseline CRP was also associated with African-American race (51% vs. 33%, *p <* 0.01) and lower income (21% vs. 11%, *p <* 0.01). Mean comorbidity score was associated with higher tertiles of each inflammatory biomarker examined. The distribution of each inflammatory biomarker are presented in Table 2; median IL-6, IL-8, and IL-10 values were 3.21, 2.63 and 9.48 pg/mL, respectively, and the median CRP value was 2.37 mg/L. The cancer types most commonly represented were lung (29%), gastrointestinal (22%), hematological (11%), and genitourinary cancers (9%).

**Table 1:** Baseline characteristics of REGARDS participants by levels of inflammatory biomarkers

	Tertiles of Inflammatory Biomarkers							
	IL-6 (pg/ml)		IL-8 (pg/ml)		IL-10 (pg/ml)		CRP (mg/L)	
	**T1**	**T3**	**T1**	**T3**	**T1**	**T3**	**T1**	**T3**
**Participants**	618	619	618	618	619	619	619	619
**Weighted Participants**	10,796	7652	9668	8533	9665	8702	9617	8347
	**Presented as Column % or Median (IQR)**							
**Age at baseline, Median (IQR)**	65 (56–72)	69 (60–76)	64 (56–72)	70 (62–76)	66 (58–75)	70 (61–76)	68 (59–76)	67 (59–75)
**African-American Race, %**	35.20	50.95	41.08	43.21	42.98	40.67	32.82	50.94
**Male Gender, %**	48.25	43.63	44.30	47.53	42.31	50.44	54.72	33.62
**Education < High School, %**	7.28	18.05	10.12	13.54	14.09	13.05	7.66	16.16
**Income <$20,000, %**	10.66	22.96	14.19	18.26	15.21	17.89	11.18	21.03
**No Exercise Activity, %**	22.22	41.80	31.45	38.51	32.94	36.67	28.00	37.62
**BMI (kg/m^2^), Median (IQR)**	26.9 (24.2–30.3)	29.1 (25.7–33.9)	28.8 (25.8–33.2)	27.9 (24.3–32.1)	28.4 (24.9–32.4)	28.3 (24.9–32.3)	26.5 (23.6–29.3)	30.1 (26.2–34.9)
**Current Smoking Status, %**	10.75	22.71	11.14	17.04	15.74	14.17	14.31	14.59
**Heavy Alcohol Consumption, %**	4.65	2.45	2.46	4.35	5.68	2.82	4.03	4.51
**Medication Use, %**								
NSAIDs - Aspirin	44.06	41.66	41.78	44.78	40.94	48.46	44.79	40.96
Statins	32.21	34.11	31.17	38.70	31.27	36.75	33.39	32.28
**Comorbid Conditions, %**								
Atrial fibrillation	10.50	8.30	6.57	10.67	7.08	9.34	8.70	8.47
Chronic lung disease	6.29	7.93	8.73	8.94	7.21	10.63	8.14	8.79
Coronary artery disease	14.79	20.98	10.38	21.76	13.31	19.86	17.76	19.36
Deep vein thrombosis	4.72	8.29	6.00	4.76	5.48	7.03	4.08	6.85
Diabetes	20.46	29.58	17.53	29.83	20.47	28.56	20.67	25.67
Dyslipidemia	54.64	59.45	54.05	65.14	54.37	64.83	55.72	60.53
Hypertension	46.44	68.44	55.24	62.76	54.23	64.79	47.82	68.48
Myocardial infarction	11.00	17.30	8.51	14.93	10.01	15.09	13.52	14.86
Peripheral artery disease	0.60	2.58	1.47	1.29	2.12	1.84	1.08	2.34
Stroke	2.74	6.38	4.01	9.53	5.19	6.66	6.45	5.22
**Comorbidity Score, Mean (SD)**	1.78 (1.39)	2.41 (1.49)	1.87 (1.41)	2.39 (1.46)	1.95 (1.46)	2.39 (1.48)	1.91 (1.43)	2.36 (1.47)

% - Denotes weighted column percentages; IQR – Interquartile range; Comorbidity score is total of comorbidities, presented as mean and standard deviation (SD). Due to statistical weighting, most *p* values are significantly <0.01.

**Table 2:** Inflammatory biomarkers distribution by tertiles

	*N*	1st Tertile	*N*	2nd Tertile	*N*	3rd Tertile	Median (IQR)
Biomarker							
IL-6 (pg/mL)	618	0.62–2.51	619	2.51–4.11	619	4.11–12.31	3.21 (2.17–4.84)
IL-8 (pg/mL)	618	0.11–2.15	620	2.16–3.25	618	3.26–281.18	2.63 (1.92–3.69)
IL-10 (pg/mL)	619	0.18–7.42	618	7.44–11.97	619	11.98–3806.61	9.48 (6.51–13.84)
CRP (mg/L)	619	0.15–1.31	618	1.32–3.93	619	3.95–111.00	2.37 (0.99–5.11)

In crude models (Table 3), every unit increase in log-transformed IL-6 was associated with a 3-fold higher risk of cancer mortality (HR: 3.15, 95% CI: 2.11–4.72) and a more than 4-fold higher risk of cancer mortality comparing the 3rd vs. 1st tertile (HR: 4.41, 95% CI: 2.45–7.95). After adjusting for age, sex, education, income, cancer site, and race, there was over a 6-fold increased risk of cancer mortality associated with the highest tertile of IL-6 (HR: 6.66, 95% CI: 2.70–16.43). The risk increased significantly to almost 13-fold after additionally adjusting for baseline health behaviors including physical activity, BMI, tobacco use, alcohol, and comorbidity score (HR: 12.97, 95% CI: 3.46–48.63). Higher levels of IL-8 were also associated with increased risk of cancer mortality after adjustment for study covariates when log-transformed (HR: 2.37, 95% CI: 1.14–4.91) and comparing the 3rd vs. 1st tertile (HR: 2.21, 95% CI: 0.86–5.71). IL-10 was associated with increased risk of cancer mortality when log-transformed (HR: 1.77, 95% CI: 1.26–2.48) and comparing 3rd vs. 1st tertile (HR: 3.06, 95% CI: 1.35–6.89) in fully adjusted models. Higher inflammatory biomarker index was associated with a 54% increased risk of overall cancer mortality (HR 1.54, 95% CI: 1.23–1.92) in fully adjusted models (Table 3). When restricted to obesity-associated cancers, IL-6 remained significantly associated with increased risk of cancer mortality (HR: 3.33, 95% CI: 1.61–6.87), but none of the other biomarkers remained significant in adjusted models (Table 4). When stratified by race (Table 5), log-transformed IL-6 was associated with almost a 4-fold increased risk among African-Americans (HR: 3.88, 95% CI: 1.17–12.88), and a 5-fold increased risk of cancer mortality among Whites (HR: 5.25, 95% CI: 1.24–22.31). There were no significant associations between CRP and cancer mortality in overall or race-stratified models.

**Table 3:** Hazard ratios (HR) and 95% confidence intervals (CI)^1^ of inflammatory biomarkers for cancer deaths (*n* = 86, weighted *n* = 1340) among all participants

	Log-Transformed	T1	T2	T3	Inflammatory Index^2^
**IL-6 (pg/mL), *N* (Cases)**		618 (16)	619 (27)	619 (43)	
**Weighted *N* (Cases)**		10,796 (279)	8449 (352)	7652 (709)	
Crude	3.15 (2.11–4.72)	Referent	2.29 (1.22–4.31)	4.41 (2.45–7.95)	1.30 (1.15–1.46)
Model 1	3.91 (2.49–6.15)	–	0.97 (0.37–2.57)	4.99 (2.30–2.57)	1.42 (1.16–1.73)
Model 2	4.70 (2.86–7.71)	–	1.31 (0.47–3.62)	6.66 (2.70–16.43)	1.46 (1.20–1.78)
Model 3	4.05 (2.25–7.31)	–	2.55 (0.72–9.05)	12.97 (3.46–48.63)	1.54 (1.23–1.92)
**IL–8 (pg/mL), *N* (Cases)**		618 (20)	620 (27)	618 (39)	
**Weighted *N* (Cases)**		9668 (233)	8697 (338)	8533 (769)	
Crude	1.45 (1.11–1.90)	Referent	1.56 (0.85–2.84)	2.21 (1.26–3.87)	
Model 1	2.00 (0.92–4.34)	–	0.92 (0.32–2.62)	2.52 (1.04–6.12)	
Model 2	2.16 (1.05–4.43)	–	0.77 (0.29–2.10)	2.63 (1.13–6.10)	
Model 3	2.37 (1.14–4.91)	–	0.60 (0.23–1.60)	2.21 (0.86–5.71)	
**IL-10 (pg/mL), *N* (Cases)**		619 (33)	618 (22)	619 (31)	
**Weighted *N* (Cases)**		9665 (482)	8530 (342)	8702 (516)	
Crude	0.96 (0.73–1.26)	Referent	0.75 (0.43–1.32)	1.12 (0.67–1.86)	
Model 1	1.52 (1.16–2.00)	–	1.46 (0.56–3.79)	1.90 (0.90–4.02)	
Model 2	1.51 (1.15–1.98)	–	1.14 (0.44–2.93)	1.86 (0.91–3.80)	
Model 3	1.77 (1.26–2.48)	–	1.87 (0.76–4.61)	3.06 (1.35–6.89)	
**CRP (mg/L), *N* (Cases)**		619 (26)	618 (30)	618 (30)	
**Weighted *N* (Cases)**		9617 (362)	8933 (499)	8347 (479)	
Crude	1.18 (0.97–1.43)	Referent	1.28 (0.74–2.20)	1.49 (0.86–2.58)	
Model 1	1.10 (0.84–1.44)	–	1.04 (0.44–2.43)	0.84 (0.38–1.89)	
Model 2	1.19 (0.90–1.59)	–	1.20 (0.50–2.88)	1.13 (0.44–2.94)	
Model 3	1.16 (0.82–1.64)	–	1.41 (0.48–4.17)	1.30 (0.45–3.77)	

^1^Model 1: Adjusted for age, gender, education, income, and site; Model 2: Additionally adjusted for race; Model 3: Additionally adjusted for physical activity, BMI, smoking status, alcohol use, and comorbidity score; T1: 1st tertile; T2: 2nd tertile; T3: 3rd tertile.

^2^ The inflammatory index is based on the summation of tertiles among four biomarkers (IL-6, IL-8, IL-10, and CRP), with scores ranging from 0 (lowest tertile in all biomarkers) to 8 (highest tertile in all biomarkers).

**Bold indicates significance at 0.05 alpha level.**

**Table 4:** Hazard ratios (HR) and 95% confidence intervals (CI) of death due to obesity-related cancers by baseline inflammatory biomarkers

	Log–Transformed
**IL-6 (pg/mL), N (Cases)**	1856 (32)
**Weighted N (Cases)**	26,897 (429)
(Crude	**3.52 (1.86–6.67)**
Model 1	**2.87 (1.44–5.69)**
Model 2	**3.33 (1.61–6.87)**
**IL-8 (pg/mL), N (Cases)**	1854 (32)
**Weighted N (Cases)**	26,898 (429)
Crude	**1.53 (1.01–2.31)**
Model 1	1.38 (0.79–2.40)
Model 2	1.38 (0.76–2.50)
**IL-10 (pg/mL), N (Cases)**	1856 (32)
**Weighted N (Cases)**	26,897 (429)
Crude	0.78 (0.46–1.35)
Model 1	0.58 (0.28–1.21)
Model 2	0.58 (0.27–1.23)
**CRP (mg/L), N (Cases)**	1856 (32)
**Weighted N (Cases)**	26,897 (429)
Crude	0.98 (0.74–1.29)
Model 1	0.97 (0.70–1.35)
Model 2	0.97 (0.69–1.37)

Model 1: Adjusted for age, gender, education, and income; Model 2: Additionally adjusted for race. Bold indicates significance at 0.05 alpha level. The obesity-related cancers included were cancers of the breast, colorectum, kidney, liver, pancreas, gastro-intestinal, endometrium, and esophagus.

**Table 5:** Hazard ratios (HR) and 95% confidence intervals (CI) of death due to obesity-related cancers by baseline inflammatory biomarkers

		African-American participants (*N* =813, weighted *N* = 11,145)		White participants (*N* =1043, weighted *N* = 15,752)	
	*P*	Log–transformed	T3	Log–transformed	T3
**IL-6 (pg/mL), N (Cases)**			304 (21)		315 (22)
**Weighted N (Cases)**			3899 (341)		3753 (368)
Crude		**3.14 (1.79–5.52)**	**6.03 (2.05–17.78)**	**3.06 (1.72–5.45)**	**3.90 (1.88–8.08)**
Model 1	0.749	**4.57 (1.44–14.49)**	4.04 (0.49–33.20)	**3.34 (1.52–7.35)**	**3.61 (1.02–12.83)**
Model 2	0.041	**3.88 (1.17–12.88)**	5.66 (0.58–55.52)	**5.25 (1.24–22.31)**	–
**IL-8 (pg/mL), N (Cases)**			300 (23)		318 (16)
**Weighted N (Cases)**			3687 (450)		4846 (320)
Crude		**2.00 (1.26–3.16)**	**2.45 (1.13–5.30)**	1.17 (0.82–1.66)	1.93 (0.85–4.38)
Model 1	0.223	0.99 (0.52–1.91)	3.93 (0.92–16.69)	2.19 (0.69–6.94)	1.83 (0.56–5.95)
Model 2	0.013	2.66 (0.54–13.13)	1.88 (0.19–18.63)	1.26 (0.21–7.72)	1.58 (0.25–9.92)
**IL-10 (pg/mL), N (Cases)**			281 (16)		338 (15)
**Weighted N (Cases)**			3539 (244)		5163 (272)
Crude		0.82 (0.53–1.27)	1.18 (0.58–2.39)	1.09 (0.82–1.45)	1.07 (0.52–2.23)
Model 1	0.119	1.08 (0.59–1.99)	0.51 (0.15–1.82)	**1.94 (1.24–3.04)**	**5.29 (1.51–18.56)**
Model 2	0.093	1.48 (0.61–3.61)	1.56 (0.23–10.53)	1.97 (0.89–4.34)	6.18 (0.94–40.39)
**CRP (mg/L), N (Cases)**			325 (18)		294 (12)
**Weighted N (Cases)**			4252 (300)		4095 (179)
Crude		1.14 (0.85–1.53)	1.50 (0.69–3.27)	1.18 (0.93–1.51)	1.31 (0.59–2.90)
Model 1	0.915	1.06 (0.70–1.60)	0.33 (0.08–1.45)	1.41 (0.79–2.52)	3.43 (0.68–17.22)
Model 2	0.426	0.92 (0.55–1.54)	0.30 (0.03–3.36)	1.59 (0.44–5.74)	–

Model 1: Adjusted for age, gender, education, income, and site; Model 2: Additionally adjusted for physical activity, BMI, smoking status, alcohol use, and comorbidity score; T3: 3rd tertile vs. 1st tertile (referent). Bold indicates significance at 0.05 alpha level. P is the *p*-value for interaction between the log of the biomarker and race. (-) Denotes unreliable parameter estimates and associated 95% confidence intervals.

## DISCUSSION

In this prospective cohort study of African-American and White participants within the REGARDS cohort, higher levels of baseline circulating IL-6 was associated with higher overall and obesity-associated cancer mortality. Higher baseline levels of IL-8 and IL-10 were also associated with cancer mortality overall but not in race stratified models. The strong associations observed for IL-6 remained statistically significant even after adjusting for socio-demographic and behavioral risk factors such as education, income, BMI, smoking, and alcohol intake, suggesting that baseline levels of this specific cytokines may independently and significantly contribute to increased mortality among cancer patients.

These findings are consistent with other studies reporting strong associations between pro-inflammatory cytokines such as IL-6, IL-8, and IL-10 with poor cancer outcomes [26–30]. Among studies evaluating cancer mortality outcomes in participants who were cancer-free at baseline, IL-6 and IL-8 were found to be important predictors of mortality. For instance, there was a 2-fold increased risk of overall cancer mortality and a 6-fold increased risk of lung cancer mortality comparing those in the highest quartiles of cytokine scores to the lowest [6]. Other studies also support the role of IL-8 in tumor initiation, proliferation, invasion, metastasis and poor prognosis [31–34]. Other studies have also evaluated the role of circulating cytokines on cancer survival among cancer patients. A recent systematic review indicated that IL-6 was inversely correlated with cancer survival in 82 studies out of 101 studies [35], and a study among breast cancer patients observed that high levels of IL-8 was correlated with a higher tumor load, metastasis, accelerated clinical course, and poorer post-relapse survival [36]. A meta-analysis of 21 studies also revealed that high IL-10 levels were associated with worse disease-free and overall survival in both solid and hematological cancer patients [37]. Other studies show significant correlations between IL-10 and aggressive tumor characteristics [38]; however, some studies reported a protective association of IL-10 on cancer survival [39, 40]. These conflicting results may be due to pleiotropic functions of IL-10 as both a pro- and anti-inflammatory cytokine.

Although we observed significant associations between IL-8 and IL-10 in the overall models, there were non-significant associations in obesity-associated cancers or race-stratified models; it is possible that reduced sample size in the sub-group analysis may have limited statistical power to detect meaningful differences. In addition, while baseline IL-6 was associated with higher cancer mortality overall and among African-Americans and Whites in this study, the association between IL-10 and cancer mortality remained significant only among Whites in our study. The majority of studies evaluating racial differences in the association between chronic inflammation and cancer outcomes have focused on survival outcomes among cancer patients. For instance, IL-6 was associated with poorer survival in both African-American and White lung cancer patients, while IL-10 and IL-12 were associated with poorer survival only among African-American lung cancer patients [41]. We did not observe a significant association between CRP and cancer mortality overall or by race, similar to other published studies using prospective cohort data [42, 43]. This study adds important information relating to the role of specific biomarkers of chronic inflammation measured at baseline with risk of cancer mortality prospectively. Results show that measures of chronic inflammation obtained many years prior to cancer death, in this case an average of 6 years between cancer-free entry into the study cohort to cancer death, may have important predictive value for cancer outcomes.

The biological mechanisms through which specific cytokines are associated with cancer mortality has been widely investigated. Th2 cytokines such as IL-6 and IL-10 enhance motility and survival of highly tumorigenic cancer stem cells and thus metastasis [44], while IL-8 is involved in the regulation of angiogenesis, cancer cell growth and survival, tumor cell motion, leukocyte infiltration, and modification of immune responses [45]. IL-6 and IL-10 are believed to influence tumor progression by activating Janus Kinase (JAK) causing the phosphorylation of signal transducer and activation of transcription 3 (STAT3), initiation and transcription of STAT3 target genes including cyclin D1, Bcl-xL, c-myc, Mcl1 and vascular endothelial growth factor (VEGF) [46]. As an immune-suppressive cytokine, IL-10 promotes tumor cell proliferation and metastasis [37, 47]. Our study suggests that if validated as a useful biomarker of cancer mortality, clinical or lifestyle strategies focusing on regulating chronic inflammation among cancer patients may be a promising cancer prevention approach, and may contribute to improved risk-stratification by identifying which patients are at higher risk of mortality and require more intensive treatment and follow-up.

The strength of this study includes the prospective design that minimized potential reverse causality where diagnosis of cancer could influence biomarker levels, and objective measures of chronic inflammatory biomarker. While we could not rule out the possibility that some latent cancers might be present at baseline, to participate in the REGARDS study, participants had to be free of cancer at baseline, so this is likely to have minimal effect. The availability of data on both African-Americans and Whites enabled stratified analysis by race; however, limited sample size in the race-stratified analyses may have limited our statistical power. The limitations of our study include potential over-representation of fatal cancers such as lung, pancreatic and ovarian cancers due the relatively short follow-up period, under-representation of more indolent breast and prostate cancers that require longer follow-up duration, and the limited number of cancer deaths that precluded analysis of site-specific mortality. In addition, we were unable to assess the association between baseline inflammatory biomarkers and cancer incidence as the REGARDS cohort was designed to examine long-term risk factors for stroke. Future studies utilizing the REGARD cohorts could aim to retrospectively collect data on incident cancer cases. We were also underpowered to detect associations between cytokines and mortality from obesity-related cancers in race-stratified analysis.

In conclusion, baseline measures of IL-6, a biomarker of chronic inflammation, and higher overall inflammatory biomarker index was associated with increased risk of cancer mortality in this racially diverse prospective cohort.

## METHODS

### Study participants

Data were obtained from the REGARDS (REasons for Geographic and Racial Differences in Stroke) cohort of 30,239 participants who were recruited between January 2003 and October 2007. Participants were aged ≥ 45 years at baseline, 45% male, 41% African-American, and 69% were >60 years old. The REGARDS cohort was originally designed to assess stroke risk between African American and non-Hispanic White Americans, and thus the study oversampled African American individuals and did not include participants of other race/ethnicities. Covariates including demographics and behavioral factors were assessed, and blood was collected at baseline as part of comprehensive physical examinations. Participants were followed-up prospectively to identify medical events, hospitalizations or deaths, and routine linkages with the national databases were used to ascertain mortality outcomes. Full details regarding the REGARDS cohort methodology has been described previously [48–50]. In the present study, a stratified random sub-cohort sample of 1,856 participants with biomarker data who were free of cancer at baseline was analyzed. The sub-cohort was selected based on a stratified random sample designed to represent the entire REGARDS cohort on race (50% African-American), sex (50% women), age (20% 45–54 years, 20% 55–64 years, 25% 65–74 years, 25% 75–84 years, and 10% ≥ 85 years), and region (30% from Stroke Belt, 20% from Stroke Buckle, and the remaining from Non-Belt regions). Stroke Belt regions are eight states in the US with higher (with average stroke mortality higher by ~20% than elsewhere in the US) stroke risk and include Alabama, Arkansas, Georgia, Louisiana, Mississippi, North Carolina, South Carolina, and Tennessee [51, 52]. Stroke Buckle regions are coastal states within the Stroke Belt with even more higher stroke mortality (about 40% higher stroke mortality than elsewhere in the US) and include Georgia, North Carolina, and South Carolina [52]. The Non-Belt regions are other contiguous states.

### Main exposure variables

In this study we examined the association between baseline circulating biomarkers for inflammation based on prior studies [26–30] and availability within the REGARDS cohort in relation to cancer mortality assessed prospectively. Biomarkers were assessed from blood samples collected during baseline physical examination, and analyzed in the REGARDS central laboratory at the University of Vermont, Burlington, VT, USA. IL-6 was measured using ultra-sensitive ELISA (Quantikine HS Human IL-6 Immunoassay, R&D Systems, Minneapolis, MN, USA); IL-8 was measured using Human Serum Adipokine Panel B LINCOplex Kit (Linco Research, Inc., St. Charles, MO, USA); and IL-10 was measured using Milliplex MAP Human Cardiovascular Disease (CVD) Panel 3 (Millipore Corporation, Billerica, MA, USA) run as a singleplex assay. A validated high-sensitivity particle-enhanced immunonephelometric assay on the BN II nephelometer (N High Sensitivity CRP, Dade Behring Inc., Deerfield, IL, USA) was used to measure CRP. The laboratory analytical intra- and inter-assay coefficient of variation (CV) for the biomarkers ranged from 1.4 to 8.1% and < 21%, respectively.

### Cancer mortality outcome

Trained REGARDS study personnel contacted participants by telephone every six-months during follow-up to identify any hospitalizations or death experienced by study participants. REGARDS investigators reviewed death certificates and interviewed proxies to delineate the primary cause of death. Further, cancer deaths occurring during follow up were identified either through death certificates, medical records, or interviewed proxies and verified through linkages with the Social Security Deaths Index (SSDI) as well as the National Death Index (NDI) following national guidelines [53]. The accuracy of SSDI and NDI has been validated before with accuracies ranging from 82–100% [54–56]. As a secondary outcome, we defined obesity-related cancer deaths as cancer deaths of the breast, colon and rectum, kidney, liver, pancreas, gastrointestinal, endometrium, and esophagus, using the International Agency for Research on Cancer definitions [57, 58]. Participants were followed-up through the date of death, or last telephone follow-up (December 31, 2012). Participants who did not die of cancer by the last date of follow-up were right censored.

### Covariates

Socio-demographic variables included in the analysis were age (continuous), sex (female/male); education (less than high school, high school, some college, or college plus), income (annual income, categorical), and race (African-American/White). Body mass index ((BMI),) was assessed during baseline physical examination. Behavioral variables included in the analysis were physical activity (none, 1–3 times/week, or ≥4 times/week), smoking (never, past, or current user), and alcohol intake (light, moderate or heavy intake). Cancer site and comorbidity score (mean number of comorbid conditions present at baseline) were also included in analysis.

### Statistical analysis

Biomarkers that were non-normally distributed were log-transformed and categorized into tertiles for analysis**.** Baseline participants characteristics were compared across tertiles of each biomarker using Chi-square and Kruskal-Wallis tests as appropriate, and the Cox proportional hazards regression with robust sandwich estimation method was used to estimate hazard ratios (HRs) and 95% confidence intervals (CIs) in sequentially adjusted models. Model 1 adjusted for baseline age, sex, education, income, and cancer site; model 2 further adjusted for race except in race stratified model; and model 3 further adjusted for baseline physical activity, BMI, smoking, alcohol, and comorbidity score. Statistical interactions between each biomarker and race were also evaluated, and race-stratified models were presented. Participants were censored at the date of death due to other reasons, loss to follow-up, or December 31, 2012, whichever happened first. SAS version 9.4 (Cary, NC, USA) was used for all statistical analysis. *P* values ≤ 0.05 were considered statistically significant and for interaction terms, *P* values ≤ 0.1 were considered statistically significant. The institutional review boards of all participating institutions approved this study.

### Sensitivity analysis

In sensitivity analysis, we created an inflammatory biomarker index based on the summation of tertiles among the four studied biomarkers (i.e., IL-6, IL-8, IL-10, and CRP). The range of this index were from zero to eight, as the lowest score a participant could have for each biomarker were zero and highest were 2 (i.e., tertile one = 0 points, tertile two = 1 point, and tertile three = 2 points). Thus, if a participant had biomarkers in the highest tertile for all four biomarkers then their respective index was eight. Likewise, if a participant had biomarkers levels within the lowest tertile for all four biomarkers then their index equaled zero. The associated HR for the inflammatory biomarker index is interpreted as a tertile (level) increase in any biomarker. We ran models with the inflammatory biomarker index as the primary exposure variable using aforementioned adjustments. We additionally fit models with all biomarkers as exposure variables.

## Abbreviations

BMI: Body Mass Index
CI: Confidence Interval
CRP: C-Reactive Protein
CV: Coefficient of variation
CVD: Human Cardiovascular Disease
HR: Hazard Ratio
IL-6: Interleukin-6
IL-8: Interleukin-8
IL-10: Interleukin-10
JAK: Janus Kinase
NDI: National Death Index
REGARDS: REasons for Geographic and Racial Disparities in Stroke
SD: standard deviation
SSDI: Social Security Death Index
STAT3: Signal transducer and activation of transcription 3
VEGF: Vascular endothelial growth factor
