# Correction: Association of baseline inflammatory biomarkers with cancer mortality in the REGARDS cohort

**DOI:** 10.18632/oncotarget.27480

**Published:** 2020-02-18

**Authors:** Tomi Akinyemiju, Justin X. Moore, Daniel Tefera Dibaba, Maria Pisu, Michael Goodman, Virginia J. Howard, Monika Safford, Susan C. Gilchrist, Mary Cushman, LeAnn Long, Suzanne E. Judd

**Affiliations:** ^1^ Department of Population Health Sciences, Duke University School of Medicine, Durham, NC, USA; ^2^ Division of Public Health Sciences and Siteman Cancer Center, Washington University School of Medicine, St. Louis, MO, USA; ^3^ Tennessee Clinical and Translational Science Institute, University of Tennessee Health Science Center, Memphis, TN, USA; ^4^ Division of Preventive Medicine, University of Alabama at Birmingham, Birmingham AL, USA; ^5^ Department of Epidemiology, Emory University Rollins School of Public Health, Atlanta, GA, USA; ^6^ Department of Epidemiology, University of Alabama at Birmingham, Birmingham AL, USA; ^7^ Department of Medicine, Weill Cornell Medical College, New York, NY, USA; ^8^ Department of Clinical Cancer Prevention and Cardiology, The University of Texas MD Anderson Cancer Center, Houston, TX, USA; ^9^ Department of Medicine and Vermont Cancer Center, Larner College of Medicine at the University of Vermont, Burlington, VT, USA; ^10^ Department of Biostatistics, University of Alabama at Birmingham, Birmingham, AL, USA

**This article has been corrected:** The third author in the listing, along with a new affiliation, has been added as follows:

**Daniel Tefera Dibaba^3^**

^3^Tennessee Clinical and Translational Science Institute, University of Tennessee Health Science Center, Memphis, TN, USA

Original article: Oncotarget. 2019; 10:4857–4867. 4857-4867. https://doi.org/10.18632/oncotarget.27108

